# Adolescents With Breakthrough COVID-19 Infections Requiring Hospitalization: A Multicenter Retrospective Study

**DOI:** 10.7759/cureus.60940

**Published:** 2024-05-23

**Authors:** Zümrüt Şahbudak Bal, Sema Yildirim Arslan, Gizem Guner Ozenen, Dicle Şener Okur, Önder Kılıçaslan, Asuman Demirbuga, Elif Afat Turgut, Nazan Dalgıc, Nursen Belet, Hatice Belkis İnceli, Aysegul Elvan-Tuz, Tugce Tural Kara, Beyhan Bulbul, Tugba Demirdag, Özlem Çakıcı, Alkan Bal, Deniz Ergun, Umut Altug, Asli Arslan, Didem Kizmaz İsancli, Selda Hancerli Torun, Ümit Çelik, Belma Yasar, İrem Ceren Erbas, Eda Karadag Oncel, Ali Akbas, Elif Gudeloglu, Semra Şen, Pelin Kacar, Elif Dede, Ercument Petmezci, Fatma Dilsad Aksoy, Adem Karbuz, Selim Öncel, Hasan Tezer, İlker Devrim, Ergin Ciftci, Mustafa Hacimustafaoglu, Zafer Kurugol

**Affiliations:** 1 Department of Pediatric Infectious Diseases, Ege University Faculty of Medicine, İzmir, TUR; 2 Department of Pediatric Infectious Diseases, University of Health Sciences Dr. Behcet Uz Child Disease and Pediatric Surgery Training and Research Hospital, İzmir, TUR; 3 Department of Pediatric Infectious Diseases, Pamukkale University, Denizli, TUR; 4 Department of Pediatric Infectious Diseases, Prof. Dr. Cemil Taşçıoğlu City Hospital, İstanbul, TUR; 5 Department of Pediatric Infectious Diseases, Istanbul University School of Medicine, İstanbul, TUR; 6 Department of Pediatric Infectious Diseases, Adana City Training Hospital, Adana, TUR; 7 Department of Pediatrics, Division of Infectious Diseases, Sisli Hamidiye Etfal Training and Research Hospital, İstanbul, TUR; 8 Department of Pediatrics, Division of Infectious Diseases, Medical School of Dokuz Eylül University, İzmir, TUR; 9 Department of Pediatrics, Division of Infectious Diseases, Medical School of Ankara University, Ankara, TUR; 10 Department of Pediatrics, Division of Infectious Diseases, Health Sciences University Tepecik Training and Research Hospital, İzmir, TUR; 11 Department of Pediatrics, Division of Infectious Diseases, Medical School of Akdeniz University, Antalya, TUR; 12 Department of Pediatrics, Division of Infectious Diseases, Medical School of Uludag University, Bursa, TUR; 13 Department of Pediatric Infectious Diseases, Gazi University, Ankara, TUR; 14 Department of Pediatrics and Child Health, Division of Pediatric Infectious Diseases, Section of Internal Medical Sciences, Faculty of Medicine, Kocaeli University, Izmit, TUR; 15 Department of Pediatric Emergency, Manisa Celal Bayar University Faculty of Medicine, Manisa, TUR; 16 Department of Pediatrics, Division of Infectious Diseases, University of Health Sciences Dr. Behcet Uz Child Disease and Pediatric Surgery Training and Research Hospital, İzmir, TUR; 17 Department of Pediatrics, Medical School of Pamukkale University, Denizli, TUR; 18 Department of Pediatrics, Division of Infectious Diseases, Medical School of Ege University, İzmir, TUR; 19 Department of Pediatrics, Division of Infectious Diseases, Prof. Dr. Cemil Taşçıoğlu City Hospital, İstanbul, TUR; 20 Department of Pediatrics, Division of Infectious Diseases, Medical School of Istanbul University, İstanbul, TUR; 21 Department of Pediatrics, Division of Infectious Diseases, Adana City Hospital, Adana, TUR; 22 Department of Pediatrics, Medical School of Akdeniz University, Antalya, TUR; 23 Department of Pediatrics, Division of Infectious Diseases, Medical School of Gazi University, Ankara, TUR; 24 Department of Pediatrics, Division of Infectious Diseases, Manisa Celal Bayar University, Manisa, TUR; 25 Department of Pediatrics, Division of Intensive Care Unit, Sisli Hamidiye Etfal Training and Research Hospital, İstanbul, TUR; 26 Department of Pediatrics, Medical School of Uludag University, Bursa, TUR; 27 Department of Pediatric Infectious Diseases, Uludag University Medical Faculty, Bursa, TUR

**Keywords:** hospitalization in covid-19, adolescent, covid-19, sars-cov-2, vaccine breakthrough infection

## Abstract

Background

Vaccines have the most important role in the battle against the COVID-19 pandemic. With the widespread use of vaccines, COVID-19 has remarkably declined. Adolescents were vaccinated after approvals for this age group, which was later than adults, and a nationwide vaccination program was implemented in August 2021 in Turkey for adolescents ≥12 years of age. Therefore, we aimed to determine the effects of the COVID-19 nationwide adolescent vaccination program on adolescent hospitalizations due to COVID-19 and multisystem inflammatory syndrome in children (MIS-C) by comparing two periods, including the vaccination period (VP) and the pre-VP (PVP). The second aim of this study is to compare the clinical features and disease severity of vaccine-breakthrough COVID-19 hospitalizations with unvaccinated individuals in the VP.

Methods

A retrospective multicenter study was conducted to determine and compare the number of hospitalizations due to COVID-19 and MIS-C between the VP (September 1, 2021, to August 31, 2022) and PVP (September 1, 2020, to August 31, 2021). We also compared the characteristics, risk factors, and outcomes of breakthrough infections of adolescents aged 12-18, which required hospitalization with the same age group of unvaccinated hospitalized individuals during the VP.

Results

During the study period, 3967 children (0-18 years) were hospitalized in the PVP and 5143 (0-18 years) in the VP. Of them, 35.4% were adolescents (12-18 years) in the PVP, and this rate was 18.6% in the VP; relative risk was 0.6467 (95% confidence interval [CI]: 0.6058-0.6904; p < 0.001). Patients with breakthrough COVID-19 were older (201 vs. 175 months, p < 0.001) and less commonly hospitalized for COVID-19 (81.5% vs. 60.4%, p < 0.001, odds ratio [OR]: 0.347 [95% CI: 0.184-0.654]). The majority of these infections were asymptomatic and mild (32% vs.72.9%: p < 0.001, OR: 5.718 [95% CI: 2.920-11.200]), and PICU admission was less frequently required (p = 0.011, OR: 0.188 [95% CI: 0.045-0.793]). Most breakthrough COVID-19 infections occurred within three months after the last vaccine dose (54.2%).

Conclusions

This study demonstrated a significant decrease in adolescent hospitalizations due to COVID-19 and MIS-C after implementing COVID-19 vaccines in Turkey. Breakthrough cases were less severe and mostly occurred three months after the last dose. This study emphasizes the importance of COVID-19 vaccines and that parents’ decisions may be changed, particularly those who hesitate to or refuse vaccination.

## Introduction

Coronavirus disease 2019 (COVID-19), caused by severe acute respiratory syndrome coronavirus 2 (SARS-CoV-2), spread worldwide, leading to more than 757 million cases and 6.8 million deaths as of February 27, 2023 [1]. SARS-CoV-2 vaccines have demonstrated both effectiveness against severe COVID-19 and decreased hospitalization due to COVID-19, representing an important milestone in decelerating the COVID-19 pandemic. Although vaccination is the most important preventive health measure against symptomatic and severe COVID-19, its effectiveness is not 100%. Therefore, breakthrough cases have been expected and reported worldwide [2,3]. In Maltezou et al.’s study of 1493 fully vaccinated healthcare workers, only four (0.3%) were hospitalized with COVID-19, none required mechanical ventilation, and the mortality rate was zero [4]. Most breakthrough infections were observed three months after the last dose of vaccine, which can be explained by decreased vaccine immunity. A case-matched control with an unvaccinated patient showed that being unvaccinated was associated with severe COVID-19 [5]. Of the 1262 breakthrough infections reported by Abu-Raddad et al. [6], only seven were severe COVID-19, and only one death occurred among 192,123 persons who received two doses of the messenger ribonucleic acid (mRNA)-1273 vaccine. Real-world data also support this finding, as a dramatic decrease in COVID-19 hospitalizations was observed after implementing SARS-CoV-2 vaccines worldwide. After the loosening of COVID-19 restrictions in Turkey, schools were opened gradually beginning on September 6, 2021, and vaccination for children and adolescents (under the age of 18) was initiated by August 2021. Increased hospitalization due to COVID-19 was observed as a result of both the school openings and the effect of the delta variant. The delta variant (B.1.617.2), which emerged as a consequence of the mutation in the spike protein of SARS-CoV-2, first appeared in India in October 2020 and was defined as a variant of concern in May 2021 [7,8]. As the delta variant was more infectious than the original strain of SARS-CoV-2, case numbers increased as it became a predominant strain [9]. A nationwide vaccination program was implemented in August 2021 in Turkey for adolescents ≥12 years of age. 

The first aim of this study is to evaluate the effects of the nationwide adolescent COVID-19 vaccination program on the number of adolescent hospitalizations due to COVID-19 and multisystem inflammatory syndrome in children (MIS-C) at 15 centers over one year and compare it with the previous year’s hospitalizations. The second aim is to compare the characteristics, risk factors, and outcomes of breakthrough SARS-CoV-2 infections in adolescents aged 12-18 that required hospitalization with unvaccinated hospitalized adolescents in the VP.

## Materials and methods

We conducted this retrospective multicenter case-control study at 15 Turkish tertiary-level hospitals, which included 9110 children hospitalized with SARS-CoV-2 polymerase chain reaction (PCR) positivity over two years. In August 2021, Turkey implemented a nationwide vaccination program for adolescents ≥12 years of age. We grouped and split the patients into two time periods: the vaccination period (VP) from September 1, 2021, to August 31, 2022, and the pre-VP (PVP) from September 1, 2020, to August 31, 2021. Between the two periods, we also compared the number of hospitalizations and the rate of 12- to 18-year-old adolescents among all hospitalized children. We grouped the hospitalized patients during the VP based on their vaccination status. Group 1 included breakthrough infections, and Group 2 included infections in unvaccinated patients. The clinical features, disease severity, and outcomes of COVID-19 were compared between the two groups. Breakthrough infection was defined in adolescents who received two doses of mRNA or inactive COVID-19 vaccines at least 14 days before infection.

A standardized questionnaire was created by our team and used to collect epidemiological data, laboratory findings, and patients’ clinical symptoms. Disease severity was categorically defined according to the World Health Organization (WHO) Clinical Progression Scale, with a score of ≥6 indicating severe disease (i.e., oxygen by non-invasive ventilation, high-flow or mechanical ventilation or vasopressors, or death) [10]. Underlying diseases, including malignant disease; transplantation recipients; chronic lung, gastrointestinal, immunodeficiency, and metabolic diseases; and immunosuppressive therapies, were recorded. The COVID-19 immunization date, type of vaccine (mRNA or inactive), and vaccine doses were also recorded. Patients were excluded from the study if they had only one dose of the COVID-19 vaccine or completed two doses of the COVID-19 vaccine <14 days before infection, if they were without vaccination information or incomplete medical records, or if they were hospitalized for an unrelated reason. The Research Ethics Committee of Ege University, İzmir, Turkey, and the Turkish Ministry of Health approved the study (ethical decision no. 22-8.1T/56). Statistical analysis was performed using IBM SPSS Statistics, version 25.0 (IBM Corp., Armonk, NY). Data were expressed as means ± standard deviations (SDs) or medians (interquartile range) for continuous variables or percentages for categorical variables. Group comparisons were made using the Student’s t-test for normally distributed data and the *Χ*^2^ test for categorical data. The Mann-Whitney U test was used to compare differences in nonparametric data between the groups. Differences and correlations were considered significant at p < 0.05.

## Results

**Figure 1:**
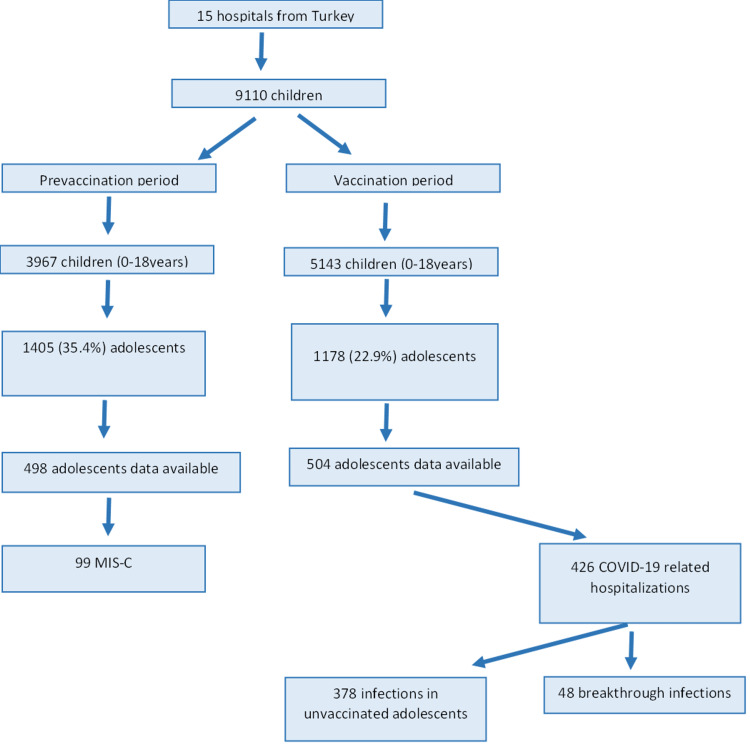
Study scheme MIS-C, multisystem inflammatory syndrome in children

During the PVP, 3967 children (0-18 years of age) were hospitalized, and 5143 were hospitalized during the VP; of them, 1405 (35.4%) and 1178 (22.9%) were adolescents, respectively (Figure 1).

Thus, adolescent hospitalization due to COVID-19 decreased in the VP (relative risk [RR]: 0.6467 [95% CI: 0.6058-0.6904]; p < 0.001). Hospitalizations due to MIS-C in this age group also significantly decreased in the VP (RR: 0.6 [95% CI: 0.4530-0.8153]; p < 0.01) (Table 1).

**Table 1:** The hospitalization and MIS-C case numbers among PVP and VP CI, confidence interval; MIS-C, multisystem inflammatory syndrome in children; PVP, pre-vaccination period; RR, relative risk; VP, vaccination period

Hospitalized patients	PVP	VP	p-value	RR; 95% CI
12- to 18-year-old hospitalizations (n)/0- to 18-year-old hospitalizations (n), [%]	1405/3967 [35.4]	1178/5143 [18.6]	<0.001	0.6467; 0.6058-0.6904
MIS-C hospitalizations (n)/0- to 18-year-old hospitalizations (n), [%]	99/ 3967 [0.025]	78/5143 [0.015]	<0.001	0.60; 0.4530-0.8153

We identified 426 hospitalized adolescents in the VP, of whom 48 (11.6%) had breakthrough COVID-19 infections. For comparison, data from only those who were hospitalized are used. Unvaccinated adolescents were significantly younger (201 vs. 175 months; p < 0.001), while no significant differences were observed in terms of gender, body mass index (BMI), length of hospital stay, or length of pediatric intensive care unit (PICU) stay (p > 0.05). Obesity was the most common underlying disease in both groups. Nephrological diseases and solid organ transplantation were significantly more common in breakthrough COVID-19 infections, with p-values of 0.030 (odds ratio [OR]: 2.5 [95% CI: 1.067-5.856]) and 0.047 (OR: 3.727 [95% CI: 1.102-12.607]), respectively. No significant differences were observed in the remaining underlying diseases. Cough and shortness of breath were less common in patients with breakthrough COVID-19, whereas abdominal pain was more common in breakthrough infections (p = 0.015, OR: 0.428 [95% CI: 0.212-0.864]); p = 0.028, OR: 0.226 [95% CI: 0.053-0.956]; and p = 0.001, OR: 3.734 [95% CI: 1.607-8.676]), respectively. Hospitalization due to COVID-19 was less common in patients with breakthrough COVID-19 (81.5% vs. 60.4%: p < 0.001, OR: 0.347 [95% CI: 0.184-0.654]), and the majority of these infections were asymptomatic and mild (32% vs. 72.9%: p < 0.001, OR: 5.718 [95% CI: 2.920-11.200]). Oxygen therapy was significantly more common in unvaccinated patients (p<0.001, OR: 0.207 [95% CI: 0.080-0.535]). Most (54.2%) breakthrough COVID-19 infections occurred at least three months after the last vaccine dose. The clinical features of breakthrough COVID-19 and the comparison with vaccinated individuals are summarized in Table 2.

**Table 2:** The clinical characteristics and risk factors of breakthrough COVID-19 infections and the comparison with unvaccinated individuals BMI, body mass index; mRNA, messenger ribonuclease; PICU, pediatric intensive care unit; SD, standard deviation; IQR, interquartile range; OR, odds ratio; CI, confidence interval; CPAP, continuous positive airway pressure; BIPAP, bi-level positive airway pressure; HFNC, high-flow nasal cannula; NA, not applicable

	Unvaccinated (n = 378)	Breakthrough (n = 48)	p-value	OR (95% CI)
Age, months, median (IQR)	175 (32.5)	201 (28.42)	<0.001	
BMI, kg/m2, median (IQR)	23.1 (7.98)	22.8 (10.69)	0.839	
Gender, male, n (%)	217 (57.4)	26 (54.2)	0.669	
Length of hospital stay, day, median (IQR)	12 (8)	20 (35)	0.605	
Length of PICU stay, day, median (IQR)	6 (7)	12 (18)	0.315	
PICU admission, n (%)	7 (18.8)	2 (4.2)	0.011	0.188 (0.045-0.793)
Underlying disease, n (%)	219 (57.9)	33 (68.8)	0.151	
Obesity, n (%)	53 (14)	7 (14.6)	0.916	
Asthma-allergy, n (%)	22 (5.8)	0 (0)	0.155	
Neurologic-neuromuscular disorder, n (%)	40 (10.6)	3 (6.3)	0.204	
Cardiovascular disease, n (%)	11 (2.9)	1 (2.1)	1	
Endocrinologic disease, n (%)	20 (5.3)	4 (8.3)	0.332	
Primary immune deficiency, n (%)	6 (1.6)	0 (0)	1	
Rheumatological diseases, n (%)	6 (1.6)	0 (0)	1	
Genetic syndrome, n (%)	14 (3.7)	3 (6.3)	0.423	
Solid malignancy, n (%)	14 (3.7)	0 (0)	0.384	
Hematologic disorder, n (%)	26 (6.9)	3 (6.3)	1	
Nephrological disease, n (%)	28 (7.4)	7 (14.6)	0.03	2.5 (1.067-5.856)
Hematologic malignancy, n (%)	21 (5.6)	2 (4.2)	1	
Pulmonary disease, n (%)	13 (3.4)	3 (6.3)	0.407	
Metabolic disease, n (%)	5 (1.3)	2 (4.2)	0.181	
Gastrointestinal system disorders, n (%)	6 (1.6)	3 (6.3)	0.069	
Solid organ transplantation, n (%)	9 (2.4)	4 (8.3)	0.047	3.727 (1.102-12.607)
Hematopoietic stem cell transplantation, n (%)	3 (0.8)	0 (0)	1	
Other, n (%)	5 (1.3)	1 (2.1)	0.514	
Severity score, mean ± SD	4.3 ± 1.5	3.4 ± 1.42	<0.001	
Symptoms, n (%)				
Fever	221 (58.5)	21 (43.8)	0.053	
Cough	155 (41)	11 (22.9)	0.015	0.428 (0.212-0.864)
Arthralgia-myalgia	40 (10.6)	2 (4.2)	0.16	
Headache	28 (7.4)	2 (4.2)	0.408	
Loss of taste and smell	8 (2.1)	0 (0)	0.606	
Fatigue	53 (14)	6 (12.5)	0.774	
Abdominal pain	22 (5.8)	9 (18.8)	0.001	3.734 (1.607-8.676)
Shortness of breath	61 (16.1)	0 (0)	0.028	0.226 (0.053-0.956)
Diarrhea	18 (4.8)	4 (8.3)	0.292	
Chest pain	25 (6.6)	2 (4.2)	0.512	
Hospitalization, n (%) COVID-19-related cause	308 (81.5)	29 (60.4)	0.001	0.347 (0.184-0.654)
Symptomatic status, n (%)				
Asymptomatic/mild disease	121 (32)	35 (72.9)	<0.001	5.718 (2.920-11.200)
Moderate/severe disease	257 (68)	13 (27.1)	<0.001	0.175 (0.089-0.343)
Fully vaccinated	16 (4.2)	48 (100)		
mRNA, n (%)	12 (75)	39 (81.25)	NA	
Inactive, n (%)	4 (25)	9 (18.75)	NA	
Non-invasive respiratory support				
Oxygen therapy, n (%)	136 (36)	6 (12.5)	<0.001	0.207 (0.080-0.535)
CPAP/BIPAP/HFNC, n (%)	46 (12.2)	1 (2.1)	0.099	
Mechanical ventilation, n (%)	15 (4)	1 (2.1)	1	
Time elapsed after vaccination, n (%)				
≤1 month		5 (10.4)	NA	
1-2 months		10 (20.8)	NA	
2-3 months		7 (14.6)	NA	
>3 months		26 (54.2)	NA	
Steroid, n (%)	112 (29.6)	10 (20.8)	0.115	
Antimicrobial therapy, n (%)	240 (63.5)	25 (52.1)	0.125	
Complications, n (%)	36 (9.5)	2 (4.2)	0.22	
Mortality, n (%)	6 (1.6)	0 (0)	1	

## Discussion

In this multicenter, retrospective, case-control study, our results showed a significant decrease in hospitalization due to COVID-19 and MIS-C among adolescents after the initiation of the COVID-19 vaccination program by the Turkish Ministry of Health compared with the previous year. Vaccinated patients were less likely to develop moderate or severe COVID-19 than unvaccinated patients and were less frequently admitted to the PICU. As expected, unvaccinated patients more frequently required oxygen therapy via nasal cannula, continuous positive airway pressure, bilevel positive airway pressure, or high-flow nasal cannula. Thus, this study adds to the literature real-life data regarding the effects of COVID-19 vaccines on hospitalizations due to COVID-19 and MIS-C. Furthermore, this is the largest and first multicenter study evaluating breakthrough infections in this age group. Tenforde et al.’s study of 4513 COVID-19 hospitalizations showed that mRNA vaccination led to a significant reduction in the requirement for mechanical ventilation and mortality among adults [11]. Since the start of the COVID-19 pandemic, older patients and those with comorbidities have carried a higher risk for morbidity and mortality caused by COVID-19. Data from the United States (US) support this finding, as children <18 years of age who had COVID-19 comprised only 1.7% of the total number of patients [12]. COVID-19 PICU surveillance data from the US show that 74 children classified as being in critical condition required PICU admission as of April 6, 2020 [13]. Therefore, vaccination for children and adolescents was pushed into the background due to limited data for this age group, and limited vaccine numbers were available. The first mRNA BNT162b2 vaccine (BioNTech, Pfizer) for administration in individuals ≥16 years of age was approved by the US Food and Drug Administration on December 11, 2020, which was amended for use in adolescents aged 12-15 years on May 10, 2021 [14]. The BNT162b2 vaccine showed a favorable safety profile, produced a sufficient immune response, and was effective against COVID-19 in recipients aged 12-15 years [15]. Subsequently, Turkey initiated vaccination for adolescents in August 2021. The decreased number of hospitalizations due to COVID-19 and MIS-C, despite the higher rate of hospitalization among adolescents compared to the previous year, supports the importance of COVID-19 vaccines in adolescents. However, vaccine hesitancy in parents plays a crucial role in the vaccination rate in this age group. In particular, reports of myocarditis associated with the mRNA vaccine among this age group have raised parents’ concerns [16]. In Bergwerk et al.’s study of 1497 fully vaccinated healthcare workers, 39 developed breakthrough COVID-19, showing milder symptoms or asymptomatic infections and lower levels of neutralizing antibodies [17]. Our results also showed that breakthrough infections were milder than those in unvaccinated patients, and vaccines significantly reduced hospitalization, PICU admission, and disease severity. However, we did not evaluate the antibody titers of our patients. In September 2021, the delta variant was dominant in Turkey and worldwide, whereas omicron became the dominant variant after December 2021. After the delta variant became prevalent, breakthrough infections increased substantially [18]. In conjunction with decreased vaccine effectiveness over time, infectivity-enhancing mutations of SARS-CoV-2 led to increased case numbers, the most dramatic of which was observed while omicron was prevalent. Our study consisted of two periods: in the first period, the alpha, beta, gamma, and delta variants of COVID-19 were prevalent. Delta was most prevalent in three months of PVP, and omicron became most prevalent after December 2021. Despite the lack of vaccination programs among adolescents during the first period of our study, total hospitalization was lower than in the PVP. This difference can be explained by the presence of COVID-19 restrictions in Turkey, which included school closures and lockdowns, as well as the relatively low infectiousness of the variants in VP. As restrictions were loosened and schools were reopened during the VP in Turkey, the number of hospitalizations increased. Despite this increase in hospitalizations, the rate of infection in adolescents was lower than in the PVP. Patients with breakthrough COVID-19 tended to have a higher BMI and were older. Obesity has been reported as one of the leading risk factors for severe COVID-19 [19]. Overall, 14.6% of breakthrough cases were obese, and a higher BMI was associated with breakthrough infection. This phenomenon can be explained by impaired glucose metabolism, even in non-obese patients. The most significant limitation of this study was its retrospective design, which may lead to missing patient data. Another limitation was the heterogeneity of vaccinated patients, as mRNA and inactive vaccines were also included in the analysis. Patients without data for their vaccination dates were excluded, which can lead to case selection bias. Finally, controls are from the PVP. It would be a better comparison if they were in the same period but not vaccinated. This study had several advantages. The first advantage was that it included 15 tertiary-level hospitals from nine cities in Turkey, which was one of the countries most affected by the COVID-19 pandemic. The large number of hospitals included and the multicenter design both increase the representativeness of the data, particularly at the national level. Finally, data on breakthrough COVID-19 hospitalizations in adolescents are scarce, and this was the largest nationwide study specifically evaluating COVID-19 severity in this age group.

## Conclusions

This study demonstrated a significant decrease in hospitalizations due to COVID-19 and MIS-C among adolescents after initiating the COVID-19 vaccination program in Turkey. Breakthrough infections were associated with older age and higher BMI. Among breakthrough infections, the severe disease was less frequently observed, oxygen therapy and other non-invasive ventilation methods were less required, and patients were less likely to be admitted to the PICU. The majority of breakthrough cases occurred three months after the last vaccine dose. This study added real-life data regarding the effectiveness of COVID-19 vaccines among adolescents. Although the COVID-19 pandemic has waned, we currently face the EG.5 (Eris) variant of SARS-CoV-2. This study emphasizes the importance of COVID-19 vaccines and may change parents’ decision-making in those who hesitate or refuse to give their children COVID-19 vaccines.
